# *Saccharomyces cerevisiae* show low levels of traversal across human endothelial barrier *in vitro*

**DOI:** 10.12688/f1000research.11782.2

**Published:** 2017-09-12

**Authors:** Roberto Pérez-Torrado, Amparo Querol

**Affiliations:** 1 Food Biotechnology Department, Institute of Agrochemistry and Food Technology (IATA-CSIC), Paterna, Valencia, Spain

**Keywords:** food emerging pathogens, Saccharomyces cerevisiae, blood brain barrier, virulence

## Abstract

**Background**: *Saccharomyces cerevisiae* is generally considered safe, and is involved in the production of many types of foods and dietary supplements. However, some isolates, which are genetically related to strains used in brewing and baking, have shown virulent traits, being able to produce infections in humans, mainly in immunodeficient patients. This can lead to systemic infections in humans.

**Methods**: In this work, we studied *S. cerevisiae* isolates in an in vitro human endothelial barrier model, comparing their behaviour with that of several strains of the related pathogens *Candida glabrata* and *Candida albicans*.

**Results**: The results showed that this food related yeast is able to cross the endothelial barrier *in vitro*. However, in contrast to *C. glabrata* and *C. albicans*, *S. cerevisiae* showed very low levels of traversal.

**Conclusions**: We conclude that using an *in vitro* human endothelial barrier model with *S. cerevisiae* can be useful to evaluate the safety of *S. cerevisiae* strains isolated from foods.

## Introduction

*Saccharomyces cerevisiae* is generally considered safe, and is involved in the production of a variety of foods and dietary supplements. Several types of food and beverage still contain viable yeast cells ^1– 5^. However, in the last years human infections with *Saccharomyces cerevisiae* have increased ^6– 8^. Consequently, *S. cerevisiae* is considered an emerging pathogen ^9– 11^. Different parts of the body can be affected in immunocompromised ^12– 15^ and healthy patients ^16– 18^. The potential virulence of this yeast has been analysed with different methods *in vitro* ^19– 22^ and *in vivo* ^23– 27^, for example by measuring epithelial barrier traversal ^28^. These reports have suggested that certain strains can cause disease and death in murine models. However, the bio-therapeutic agent Ultralevure ( *S. cerevisiae* var. *boulardii*) and other supplements are consumed in high doses, ranging from 10 ^7^ to 10 ^10^ live yeast cells per day and for long periods.

The study of yeast virulence includes studying their behaviour when they encounter endothelial barriers. Opportunistic pathogenic yeasts such as *C. glabrata* and *C. albicans* are able to pass the intestinal barrier ^29, 30^ and generate systemic infections ^31– 33^. Also, *C. albicans* can cross the blood-brain barrier (BBB) to reach the brain ^34, 35^. Regarding *S. cerevisiae,* infections after oral ingestion ^16^ or digestive translocation ^12, 14, 36^ show that it can reach brain in murine models ^25^. However, few studies have investigated the behaviour of *S. cerevisiae* when they reach endothelial barriers ^28^.

## Methods

### Yeast strains and growth media

The yeast strains are described in Table 1. Strains were propagated in YPD media (1% glucose, 1% BactoPeptone, 0.5% yeast extract) for 24 h at 30°C.

**Table 1.** Yeast strains used in this study.

Strain	Species	Source
W303	*S. cerevisiae*	From our collection
102	*S. cerevisiae*	Vall d’Hebron Hospital (Barcelona, Spain) ^19^
60	*S. cerevisiae*	Vall d’Hebron Hospital (Barcelona, Spain) ^19^
Cb	*S. cerevisiae*	Vall d’Hebron Hospital (Barcelona, Spain) ^19^
Co	*C. glabrata*	Vall d’Hebron Hospital (Barcelona, Spain)
C2	*C. glabrata*	Provided by B. Hube (Friedrich Schiller University; Jena, Germany)
C4	*C. glabrata*	Provided by B. Hube (Friedrich Schiller University; Jena, Germany)
C5	*C. glabrata*	Provided by B. Hube (Friedrich Schiller University; Jena, Germany)
CA-1	*C. albicans*	Statens Serum Institute (Copenhagen, Denmark)
SC5314	*C. albicans*	Provided by A. Yañez ^22^ (Universitat de Valencia, Spain)
ATCC26555	*C. albicans*	Provided by A. Yañez ^22^ (Universitat de Valencia, Spain)
CBS562	*C. albicans*	From our collection

### Growth of mammalian cells

Human umbilical endothelial cells (HUVECs) (Clonetics®) were grown in minimum essential medium (Earle’s salt, 25 mM HEPES and GlutaMAX™, Invitrogen) supplemented with 10% foetal bovine serum (FBS, Cambrex Bio Science), 1% nonessential amino acids (Invitrogen) and 50 μg mL ^–1^ gentamicin (Invitrogen). The cells were grown in 150 cm ^2^ culture flasks (TPP) at 37°C in a humidified atmosphere of 5% CO _2_ and 95% air until a confluence. Culture medium was changed every second day.

### Trans-epithelial electrical resistance (TEER) assay

HUVEC cells (1×10 ^5^ cells/cm ^2^) were seeded on Transwell® filter inserts (8 μm, Corning Incorporated) in 24-well plates (Corning Incorporated). A volume of 200 μL cell growth medium was added to the apical compartment and 1250 μL to the basolateral compartment. The TEER was measured using the Millicell-ERS Electrical Resistance System (Millipore). The net value of the TEER (Ωcm ^2^) was corrected for background resistance by subtracting the contribution of the cell-free filter and the medium (110 Ωcm ^2^). The TEER was measured before the addition of yeasts.

### Determination of permeability coefficient

1 μg/mL of fluorescein (Sigma) was added to the media in the apical compartment of the transwell, with or without established HUVEC monolayers, and fluorescence was measured over time in the media of the apical and basolateral compartment. The apparent permeability, Papp, was defined as (Hilgers *et al*., 1990):

*Papp* = (Δ *A _R_*/Δt))/ *C*_D,0_                                                                                                (1)

(ΔA _R_/Δt) is the rate of drug appearance in the receiver side, S is the surface area of the Transwell (0.33 cm ^2^ for Transwell® inserts (8 μm pore size, Corning) of 6.5-mm insert diameter), and C _D,0_ is the initial drug concentration in the donor side at time = 0. Values are expressed in cm/s.

### Ability to cross the endothelial barrier

HUVEC cells were seeded on Transwell® filter as described above. Yeasts grown overnight at 30°C in YPD were resuspended (10 ^6^ cells mL ^–1^) in the apical compartment and incubated at 37°C in a humidified atmosphere of 5% CO _2_ and 95% air. After 12 h, the basolateral compartment medium was replaced. Colony forming units were counted in YPD plate triplicates after two days. Control wells used to evaluate yeast growth showed no significant growth after 12 h. Negative control HUVEC Transwells without adding cells were performed to control TEER stability across the experiment.

## Results

### Evaluation of the endothelial barrier integrity

To establish an *in vitro* human endothelial barrier, we used HUVEC monolayers, a methodology that has been widely used ^37, 38^. Monolayers were formed in transwells and two different methods were used to determine the robustness, consistency and integrity of the barrier. First, we studied the TEER, indicative of physical separation. After seeding the HUVECs, TEER was measured and we observed increased values over time that were overcoming 450 Ωcm ^2^, which correlates with the establishment of a monolayer barrier. Second, we studied the monolayer permeability. The value obtained was 1.82±0.13 (10 ^-6^ cm/s) on average, which indicates an integral barrier with low permeability ^39^.

### Study of the ability of yeast species to cross the human endothelial barrier *in vitro*

**Figure 1.**
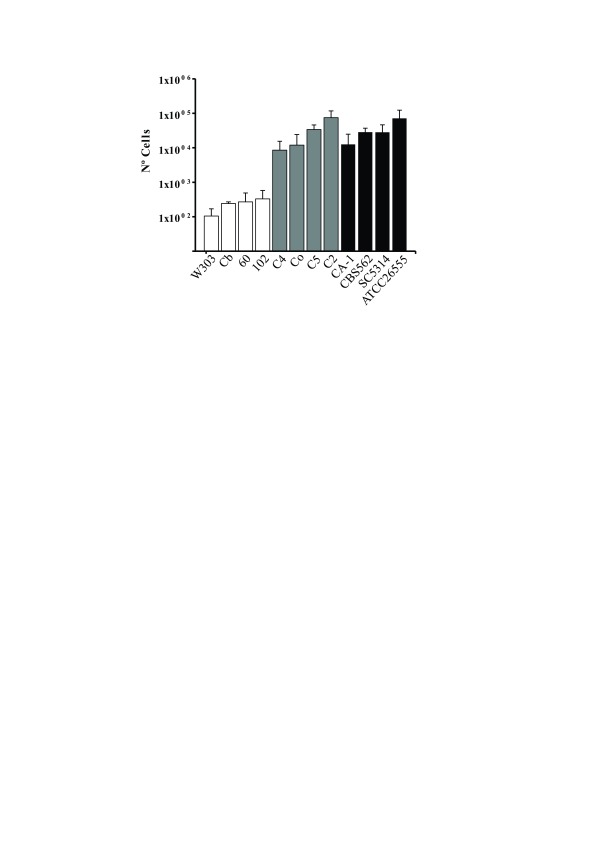
Number of yeast cells that were able to cross the endothelial barrier. To perform this assay we established HUVEC monolayers in Transwell® filter inserts in 24 well plates. 24 hours after apical addition of various strains of *S. cerevisiae*, *C. albicans* and *C. glabrata*, yeast cells from the basolateral compartment were incubated on YPD plates and colonies were counted after one day of growth. Values were obtained after plating several dilutions of the basolateral compartment media. Average of three experiments and standard deviation is shown. To determine statistically significant data, Student *t*-tests were performed in Excel with 0.05 as the *p*-value.

To determine whether *S. cerevisiae* is able to cross the human endothelial barrier, we used an *in vitro* model of the endothelium with HUVECs ^40^. The number of cells in the basolateral compartment was measured 12 hours after addition of *S. cerevisiae*, *C. albicans* and *C. glabrata* strains to the apical compartment ( Figure 1). The results showed that all yeast strains were able to cross the endothelial barrier. While elevated number of cells from *C. glabrata* and *C. albicans* strains were able to cross the endothelial barrier, *S. cerevisiae* values were low. Furthermore, while the *S. cerevisiae* control strain W303 showed the lowest levels of yeast transcytosis, the other opportunistic pathogenic strains presented higher levels.

**Figure 2.**
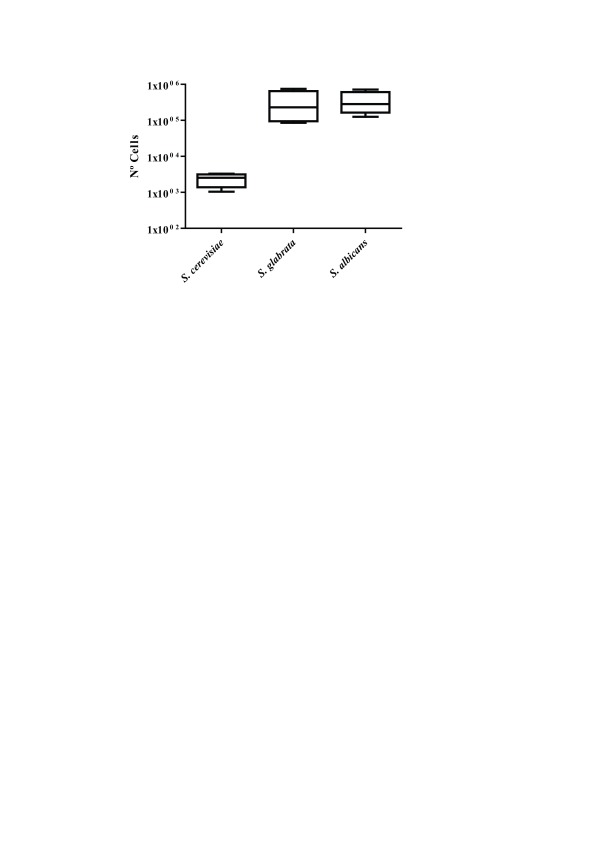
Box graph comparing the number of cells able to cross the endothelial barrier in the three yeast species.

To compare the different species, the average level of cell transcytosis for all strains of each species was calculated ( Figure 2). After 12 h, *Candida* species showed a high number of cells in the basolateral chamber (4.9–5.7 Log _10_ units). On the contrary, we observed that *S. cerevisiae* showed significantly lower levels (1.0–3.3 Log _10_ units) than the *Candida* species.

Raw data of permeability measurements and cell counts for endothelium traversal Click here for additional data file. Copyright: © 2017 Pérez-Torrado R and Querol A 2017 Data associated with the article are available under the terms of the Creative Commons Zero "No rights reserved" data waiver (CC0 1.0 Public domain dedication).

## Discussion

A model for traversal across the e *in vitro* has been used to study behaviour and pathogenicity mechanisms of yeast strains such as *C. albicans* ^34, 35^. Here, we have shown that *S. cerevisiae* strains are able to cross the endothelial barrier. This data is in accordance with previous studies, where *S. cerevisiae* cells were observed in the brain after systemic infections in murine models ^25^. When comparing to other well-known yeast pathogens such as *C. glabrata* and *C. albicans*, none of the *S. cerevisiae* strains were able to cross the endothelial barrier at high levels. Despite *S. cerevisiae* pathogenicity levels being lower than other opportunistic yeasts, we recommend the potential risk of new *S. cerevisiae* strains to be evaluated before using them in food production.

## Data availability

The data referenced by this article are under copyright with the following copyright statement: Copyright: © 2017 Pérez-Torrado R and Querol A

Data associated with the article are available under the terms of the Creative Commons Zero "No rights reserved" data waiver (CC0 1.0 Public domain dedication). 

Dataset 1: Raw data of permeability measurements and cell counts for endothelium traversal. DOI, 10.5256/f1000research.11782.d177554 ^41^
