# Supplementary material for: Saccharomyces cerevisiae show low levels of traversal across human endothelial barrier in vitro
Source: F1000Res. 2017 Sep 12;6:944. Originally published 2017 Jun 20. [Version 2] doi: 10.12688/f1000research.11782.2 (PMC5605952; doi:10.12688/f1000research.11782.2)
Supplement: Peer review history

# Peer review: Referee response for version 2

**DOI:** 10.5256/f1000research.13710.r25924

**Published:** 2017-09-19

**Authors:** Felipe H. Santiago-Tirado

**Affiliations:** 1 Department of Molecular Microbiology, Washington University School of Medicine, St. Louis, MO, USA

By stating that their model is an endothelial barrier *in vitro* model rather than a blood-brain barrier model, the authors have addressed my main concern. Considering that they submitted this work as a 'Note', defined as a small, often preliminary study, I believe it is suitable for indexing at F1000. I, nevertheless, encourage them to consider my previous minor comments on their follow up studies.

I have read this submission. I believe that I have an appropriate level of expertise to confirm that it is of an acceptable scientific standard.

# Peer review: Referee response for version 1

**DOI:** 10.5256/f1000research.12728.r25622

**Published:** 2017-09-01

**Authors:** Felipe H. Santiago-Tirado

**Affiliations:** 1 Department of Molecular Microbiology, Washington University School of Medicine, St. Louis, MO, USA

Although the article is interesting and the data clear, I believe the authors are overstating the findings. First, HUVECs are not considered a good model for blood-brain barrier anymore. They used to be a favorite one because they are a human cell line, however, they are not of cerebral origin, and deviate considerably from the behavior of cerebral endothelial cells. They could fix this by calling their model an "endothelial" monolayer instead, or repeat the experiment using "real" BBB cell lines (i.e. hCMEC/D3, which is commercially available). Also, they report the TEER values (which by the way the correct units should be resistance (ohms) times area (cm ^2^) rather than dividing by it) before the start of the experiment, but they should also measure the integrity of the monolayer at the end of the experiment, to rule out that the amount of *S. cerevisiae* crossing is due to rupture of the monolayer. This assay is also hard to interpret in the absence of a negative control - in fact, *S. cerevisiae* has been traditionally used as a negative control on this type of assays! Would inert beads also cross? Would any other organisms cross at the same rate? Maybe they can check this by using fluorescent beads and measuring fluorescence on the bottom. Or if easier to do by CFUs, they could add another organism known to not been able to cross and count CFUs. Overall, it is a nice preliminary report, one worth the time pursuing. Considering this was submitted as a Research Note, I believe is appropriate for indexing once they address my comments above.

I have read this submission. I believe that I have an appropriate level of expertise to confirm that it is of an acceptable scientific standard, however I have significant reservations, as outlined above.

# Peer review: Referee response for version 1

**DOI:** 10.5256/f1000research.12728.r24594

**Published:** 2017-08-14

**Authors:** Rosa de Llanos

**Affiliations:** 1 School of Applied Sciences, Edinburgh Napier University, Edinburgh, UK

1. Introduction:
  I would consider to change the sentence “Consequently, S, cerevisiae is considered an emerging pathogen” with “Consequently, S, cerevisiae is considered an emerging pathogen **of low virulence**”
2. Origin of isolation of the yeast strains could be included in Table 1.
3. Methods:
  Abbreviation of BBB should be added in the title Ability to cross the blood-brain barrier.
4. Results:
  In Figure 1 there are different colours but not information about the meaning of it has been included. In Figure 2, there is a mistake for C. glabrata and C. albicans, they are named as S.glabrata and S.albicans.

I have read this submission. I believe that I have an appropriate level of expertise to confirm that it is of an acceptable scientific standard.
